# Community burden and prognostic impact of reduced kidney function among patients hospitalized with acute decompensated heart failure: The Atherosclerosis Risk in Communities (ARIC) Study Community Surveillance

**DOI:** 10.1371/journal.pone.0181373

**Published:** 2017-08-09

**Authors:** Kunihiro Matsushita, Lucia Kwak, Noorie Hyun, Marina Bessel, Sunil K. Agarwal, Laura R. Loehr, Hanyu Ni, Patricia P. Chang, Josef Coresh, Lisa M. Wruck, Wayne Rosamond

**Affiliations:** 1 Johns Hopkins Bloomberg School of Public Health, Baltimore, Maryland, United States of America; 2 University of North Carolina, Chapel Hill, North Carolina, United States of America; 3 Mount Sinai Health Systems, New York City, New York, United States of America; 4 Centers of Disease Control and Prevention, Atlanta, Georgia, United States of America; 5 Duke Clinical Research Institute, Durham, North Carolina, United States of America; Nagoya University, JAPAN

## Abstract

**Background:**

Kidney dysfunction is prevalent and impacts prognosis in patients with acute decompensated heart failure (ADHF). However, most previous reports were from a single hospital, limiting their generalizability. Also, contemporary data using new equation for estimated glomerular filtration rate (eGFR) are needed.

**Methods and results:**

We analyzed data from the ARIC Community Surveillance for ADHF conducted for residents aged ≥55 years in four US communities between 2005–2011. All ADHF cases (n = 5, 391) were adjudicated and weighted to represent those communities (24,932 weighted cases). The association of kidney function (creatinine-based eGFR by the CKD-EPI equation and blood urea nitrogen [BUN]) during hospitalization with 1-year mortality was assessed using logistic regression. Based on worst and last serum creatinine, there were 82.5% and 70.6% with reduced eGFR (<60 ml/min/1.73m^2^) and 37.4% and 26.6% with severely reduced eGFR (<30 ml/min/1.73m^2^), respectively. Lower eGFR (regardless of last or worst eGFR), particularly eGFR <30 ml/min/1.73m^2^, was significantly associated with higher 1-year mortality independently of potential confounders (odds ratio 1.60 [95% CI 1.26–2.04] for last eGFR 15–29 ml/min/1.73m^2^ and 2.30 [1.76–3.00] for <15 compared to eGFR ≥60). The association was largely consistent across demographic subgroups. Of interest, when both eGFR and BUN were modeled together, only BUN remained significant.

**Conclusions:**

Severely reduced eGFR (<30 ml/min/1.73m^2^) was observed in ~30% of ADHF cases and was an independent predictor of 1-year mortality in community. For prediction, BUN appeared to be superior to eGFR. These findings suggest the need of close attention to kidney dysfunction among ADHF patients.

## Introduction

Reduced kidney function is a strong predictor of adverse health outcomes in a broad range of populations [1–6]. Patients with heart failure (HF) are no exception, and various studies have reported that low kidney function is associated with poor prognosis in this population as well [7–21]. However, many of these studies investigate patients with compensated chronic HF [7–15] and data on patients with acute decompensated HF (ADHF) are relatively limited [16–21]. More importantly, those studies with ADHF patients are from a clinical trial with stringent inclusion criteria [16] or from a single hospital [17–19]. Thus, the prevalence of kidney dysfunction and its prognostic impact among ADHF patients in the community are yet to be investigated. Though a report from a large registry study in the US [20, 21] may more reflect community setting than trials or single center studies, data completeness and lack of validation of ADHF cases remain important challenges [22]. Furthermore, many of above studies explored serum creatinine levels or creatinine clearance but not necessarily the best overall measure of kidney function recommended in clinical guidelines, estimated glomerular filtration rate (eGFR) [23]. Also, contemporary data are warranted since new and more valid equations for estimating GFR are currently available [24, 25].

In above contexts, the aims of this study were to evaluate the prevalence of reduced eGFR assessed by the contemporary creatinine-based CKD-EPI equation [25, 26] and its prognostic impact among patients hospitalized for acute decompensated HF (ADHF) in community hospitals using the community surveillance data from the Atherosclerosis Risk in Communities (ARIC) [27]. We also evaluated whether the impact of kidney dysfunction was consistent across subgroups according to age, sex, and race and contrasted the prognostic impact of eGFR and blood urea nitrogen (BUN), another parameter of kidney function demonstrating strong prognostic value in patients with ADHF [20, 21].

## Methods

### Study design and population

Details of the ARIC Community Surveillance Study have been provided elsewhere [27, 28]. Briefly, beginning in 2005, the ARIC Study performed continuous community surveillance of hospitalized ADHF for all residents aged ≥55 years in four US communities: Forsyth County, North Carolina; Jackson, Mississippi; Minneapolis, Minnesota; and Washington County, Maryland. A stratified random sample of eligible hospitalizations for HF was selected according to the following International Classification of Diseases, 9th Revision, Clinical Modification (ICD-9-CM) discharge diagnosis codes in any position: rheumatic heart disease (398.91), hypertensive heart disease with HF (402.01, 402.11, 402.91), hypertensive heart disease and renal failure with congestive HF (404.01, 404.03, 404.11, 404.13, 404.91, 404.93), cor pulmonale (415.0), unspecified chronic pulmonary heart disease (416.9), other primary cardiomyopathies (425.4), HF (428.x), unspecified acute edema of lung (518.4), and dyspnea and respiratory abnormalities (786.0x). The sampling probabilities varied by ICD-9-CM codes (428 and non-428), field center, gender, and race to optimize variance estimates across these groups.

Hospital records of eligible HF cases were abstracted by trained abstractors if there is any evidence of decompensation or new onset of HF symptoms or any mention by a physician that HF was the reason for hospitalization in the medical record. Fully abstracted cases were classified by computer algorithm or two physicians of the ARIC Mortality and Morbidity Classification Committee as definite ADHF, possible ADHF, chronic stable HF, HF unlikely, or unclassifiable based on symptoms, signs from physical examination, laboratory, and imaging, and HF-specific treatment. None of authors had direct access to information that could identify individuals. Disagreements were adjudicated by the Committee chair. For this study, definite and possible cases were considered as ADHF. Between 2005 and 2011, 5,946 ADHF cases were identified out of 15,660 eligible cases. Of these, we excluded cases other than black or white or with missing variables of interest (n = 555), leaving the study sample of 5,391 unweighted cases corresponding to 24,932 cases in those four communities.

### Exposures and covariates

All data on exposures and covariates were obtained from detailed abstraction of the medical record. For laboratory measurements, ARIC systematically recorded their worst (e.g., highest value for serum creatinine and BUN and lowest value for hemoglobin and sodium) and last values during hospitalization. As recommended in clinical guidelines [23], kidney function was primarily evaluated by eGFR, which was calculated using CKD-EPI equation with the worst and last serum creatinine, respectively [25, 26]. We also analyzed BUN, as an alternative kidney dysfunction marker, since its prognostic value has been demonstrated in patients with ADHF [20, 21]. Covariates of interest were age, race, gender, current smoking status, health insurance type (Medicare/Medicaid, private, or others), diabetes, hypertension, history of coronary heart disease, HF type (preserved vs. reduced ejection fraction [< vs. ≥50%]), history of chronic obstructive pulmonary disease, hemoglobin, and serum sodium.

### Mortality outcomes

Mortality status over 1 year after ADHF admission was identified through the linkage to the National Death Index. Although the primary outcome was 1-year mortality, we also assessed in-hospital mortality and 28-day mortality.

### Statistical analysis

All analyses were weighted by sampling fractions specific to each of the ARIC communities, accounting for population size, sex, and race. Baseline characteristics were compared across clinical eGFR categories, ≥60, 45–59, 30–44, 30–15, and <15 ml/min/1.73m^2^ or on dialysis [23]. Cumulative survival in every eGFR category was illustrated using Kaplan-Meier estimates. Since two of three follow-up periods in this study were short, we used logistic regression models to quantify the association of kidney function with mortality after accounting for the covariates listed above. We separately adjusted for hemoglobin and sodium, since they can be mediators linking reduced kidney function to poor prognosis. We also tested separate models simultaneously incorporating both kidney parameters of interest, eGFR and BUN. For a fair comparison, BUN was categorized into five groups corresponding to the percentiles based on clinical eGFR categories shown above. As both worst and last eGFR have respective aspects potentially leading to poorer outcomes than the other (i.e., worst may account for deterioration during hospitalization such as acute kidney injury, and last may reflect conditions chronologically closest to outcomes), we basically analyzed them in parallel. We repeated our analysis in demographic subgroups and tested interaction based on likelihood ratio test.

We also assessed whether evidence-based HF treatment such as beta-blockers, renin-angiotensin system inhibitors, and diuretics was provided at discharge differently across eGFR categories [29] and, if so, whether treatment status modified the association of kidney function with mortality. For this specific part of analysis, as the length of stay may reflect disease severity and thus may influence indication of HF-specific medications, we restricted to cases that were discharged alive within 28 days. Given that the medications were assessed at discharge, we focused on last eGFR for this analysis. All statistical analyses were conducted with SAS (SAS Institute Inc. Cary, North Carolina).

## Results

Of the weighted 24,932 ADHF cases, there were 70.6% and 82.5% cases with reduced eGFR (<60 ml/min/1.73m^2^) based on last and worst serum creatinine, and 26.6% and 37.4% with severely reduced eGFR (<30 ml/min/1.73m^2^), respectively (Table 1 and S1 Table). Those with reduced eGFR were more likely to be women, white, and older and to have hypertension, diabetes, and coronary heart disease compared to those with preserved eGFR. In contrast, those with reduced eGFR were unlikely to be current smoker and have history of chronic obstructive pulmonary disease.

**Table 1:** Baseline characteristics according to eGFR categories based on the last serum creatinine levels during ADHF hospitalization.

Baseline characteristics	eGFR:60+	eGFR:60–45	eGFR:45–30	eGFR:30–15	eGFR<15
Weighted N	7329 (29.4)	5295 (21.2)	5679 (22.8)	3934 (15.8)	2696 (10.8)
Age	72 (22.5)	76 (22.7)	78 (22.1)	79 (20.2)	73 (21.1)
Female	3526 (48.1)	2848 (53.8)	3278 (57.7)	2154 (54.8)	1357 (50.3)
African-Americans	2404 (32.8)	1342 (25.3)	1380 (24.3)	1032 (26.2)	1112 (41.2)
Smoker	1439 (19.6)	711 (13.4)	544 (9.6)	332 (8.4)	331 (12.3)
Hypertension	5943 (81.1)	4422 (83.5)	4892 (86.1)	3379 (85.9)	2427 (90.0)
Diabetes	3196 (43.6)	2209 (41.7)	2621 (46.2)	2207 (56.1)	1694 (62.8)
Health Insurance					
Medicare/Medicaid	1997 (29.0)	1482 (29.2)	1614 (29.2)	1093 (28.7)	1043 (40.1)
Private	4887 (71.0)	3591 (70.8)	3921 (70.8)	2710 (71.3)	1559 (59.9)
History of HF	4634 (63.2)	3759 (71.0)	4214 (74.2)	2983 (75.8)	2082 (77.2)
Prior HF hospitalization	2300 (31.4)	1750 (33.1)	2198 (38.7)	1459 (37.1)	1162 (43.1)
Prior HF treat	3760 (51.3)	3216 (60.7)	3611 (63.6)	2542 (64.6)	1727 (64.1)
Ejection fraction					
Preserved	2936 (44.0)	2052 (42.5)	2362 (45.2)	1482 (42.7)	1040 (45.5)
Reduced	3737 (56.0)	2773 (57.5)	2859 (54.8)	1987 (57.3)	1244 (54.5)
COPD	2870 (39.2)	1942 (36.7)	1975 (34.8)	1213 (30.8)	851 (31.6)
Coronary Disease	3145 (42.9)	2421 (45.7)	2785 (49.0)	1896 (48.2)	1257 (46.6)
Hemoglobin	12 (1.9)	12 (1.9)	11 (1.8)	11 (1.5)	11 (1.7)
Sodium	139 (3.9)	139 (3.6)	139 (3.9)	139 (4.5)	138 (4.5)
BUN	20 (9.6)	26 (11.3)	35 (15.7)	52 (21.1)	52 (28.9)
Systolic BP	142 (31.4)	142 (33.2)	142 (33.2)	140 (34.4)	145 (37.9)
Diastolic BP	79 (20.0)	78 (19.6)	76 (20.0)	75 (20.0)	77 (20.8)

Values are mean (SD) and n (%)

**Fig 1:**
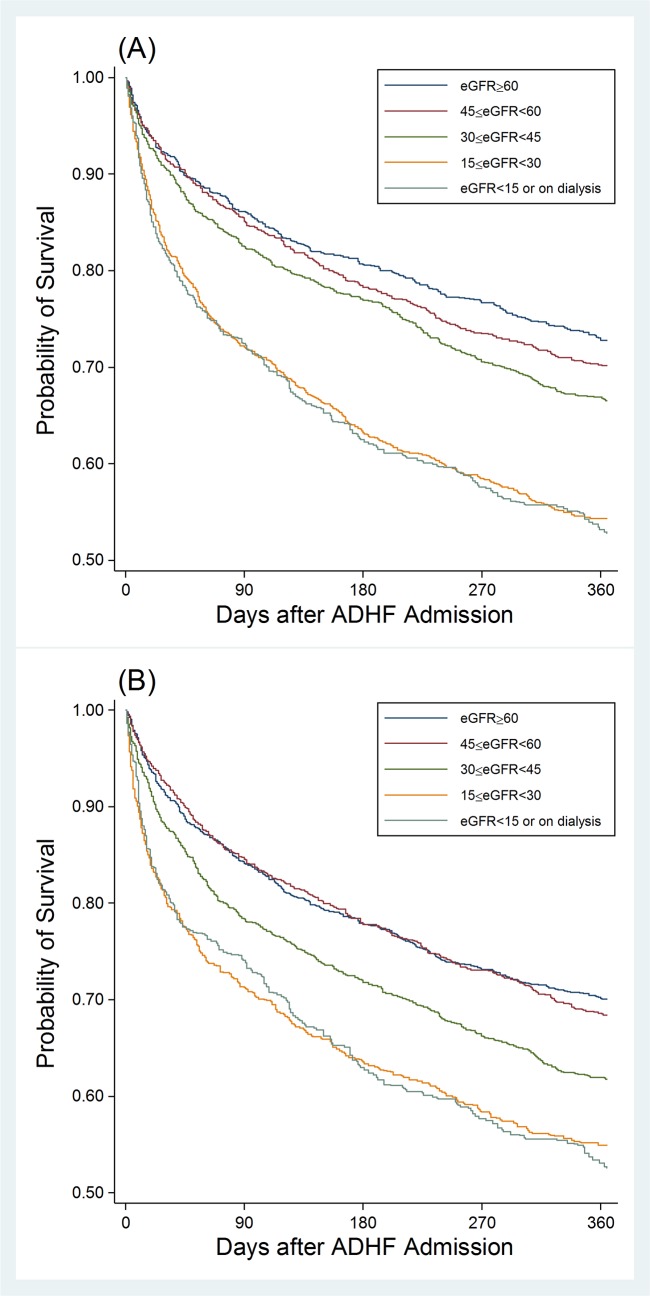
1-year survival according to eGFR categories based on the worst (A) and last (B) serum creatinine after ADHF admission.

**Fig 2:**
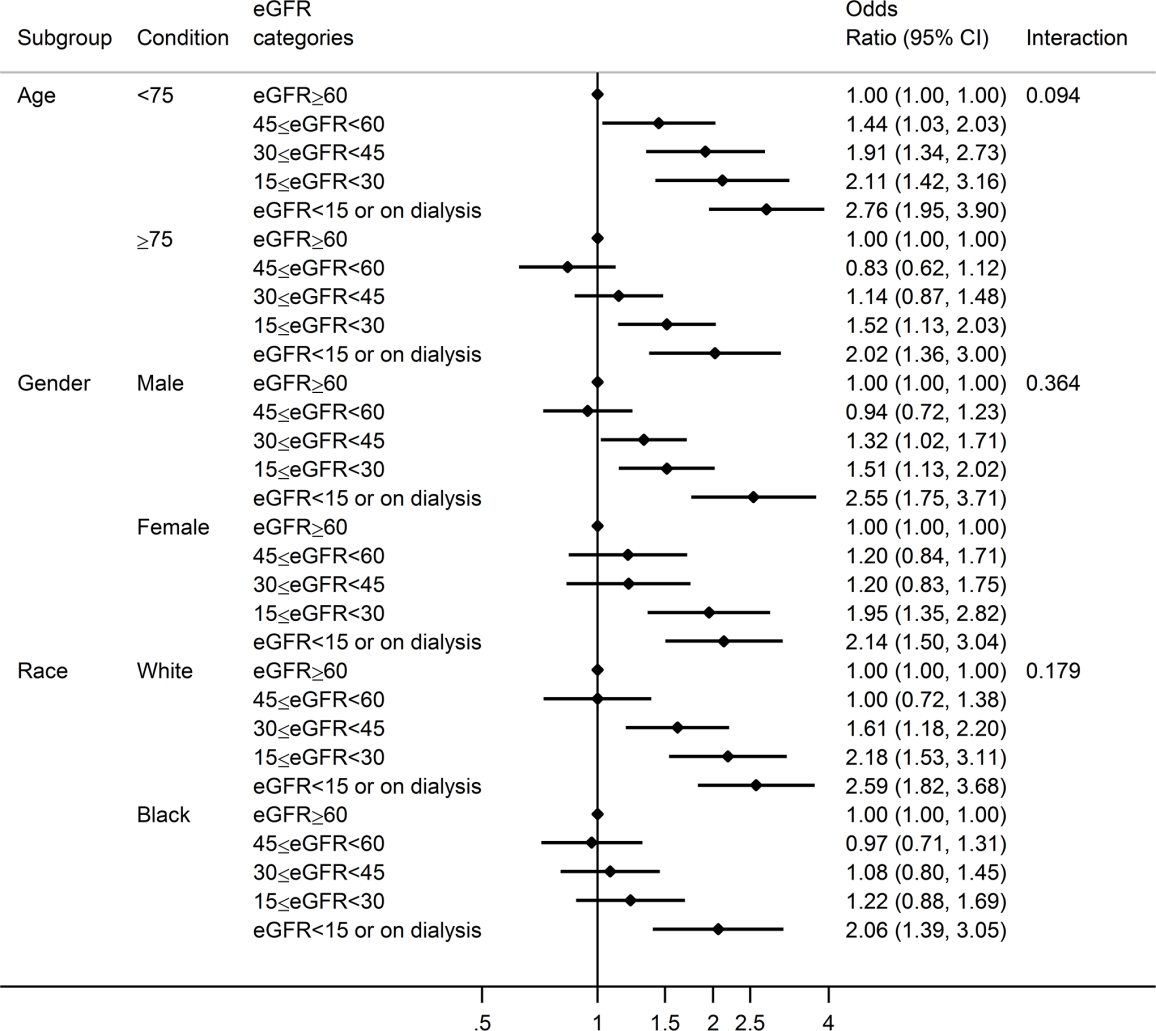
Adjusted odds ratio of 1-year mortality according to eGFR categories based on last serum creatinine in demographic subgroups. Models adjusted for primary covariates: age, race, gender, health insurance, diabetes, hypertension, smoke, history of coronary heart disease, heart failure type (preserved vs. reduced ejection fraction), and chronic obstructive pulmonary disease.

Regardless of whether we used worst or last serum creatinine, patients with severely reduced eGFR <30 ml/min/1.73m^2^ demonstrated evidently poor prognosis, with 1 year survival close to 50% (Fig 1). eGFR ≥60 ml/min/1.73m^2^ consistently demonstrated most favorable 1-year prognosis, but its separation with eGFR categories 45–59 (CKD stage 3a) was not evident with last eGFR (Fig 1B). The associations of eGFR <30 ml/min/1.73m^2^ with poor prognosis were significant even after accounting for key potential confounders for both worst and last eGFRs (Table 2). The effect size tended to be larger for short-term mortality (i.e., in-hospital and 28-day) compared to 1-year mortality. The results were overall comparable between worst and last eGFRs, although eGFR 30–44 ml/min/1.73m^2^ reached significance only with last eGFR for 1-year mortality. The association was qualitatively consistent across demographic subgroups, without any significant interactions (Fig 2).

**Table 2:** Adjusted odds ratio of mortality outcomes according to eGFR categories.

Mortality	eGFR categories				
	≥60	45–59	30–44	15–29	<15/dialysis
			Worst		
In-hospital	reference	0.96 (0.53, 1.75)	1.11 (0.64, 1.91)	2.04 (1.19, 3.48)	4.79 (2.88, 7.95)
28-day	reference	1.14 (0.73, 1.79)	0.99 (0.66, 1.51)	2.09 (1.40, 3.12)	3.42 (2.27, 5.15)
1-year	reference	1.05 (0.81, 1.36)	1.26 (0.99, 1.61)	1.96 (1.53, 2.53)	2.58 (1.96, 3.38)
			Last		
In-hospital	reference	0.72 (0.44, 1.18)	1.22 (0.79, 1.90)	2.10 (1.35, 3.26)	4.99 (3.30, 7.56)
28-day	reference	0.87 (0.59, 1.27)	1.25 (0.89, 1.77)	2.06 (1.45, 2.92)	3.66 (2.55, 5.25)
1-year	reference	0.99 (0.79, 1.24)	1.29 (1.04, 1.60)	1.60 (1.26, 2.04)	2.30 (1.76, 3.00)

Adjusted for primary covariates: age, race, gender, health insurance, diabetes, hypertension, smoke, history of coronary heart disease, heart failure type (preserved vs. reduced ejection fraction), and chronic obstructive pulmonary disease.

Further adjustment for prespecified potential mediators, i.e., hemoglobin and sodium, did not alter the association between reduced kidney function and mortality (Table 3). On the other hand, when BUN was included in the model, eGFR was no longer significantly associated with increased mortality (Table 3). In contrast, BUN, regardless of its worst (highest) or last value, was significantly associated with 1-year mortality even after accounting for eGFR as well as other potential confounders (Table 4).

**Table 3:** Adjusted odds ratio of 1-year mortality according to eGFR categories with additional adjustment for potential mediators (sodium and hemoglobin) and another kidney measure, blood urea nitrogen (BUN).

			eGFR categories		
Models including primary covariates plus	≥60	45–59	30–44	15–29	<15/dialysis
			Worst		
Hemoglobin	reference	1.02 (0.78, 1.33)	1.16 (0.90, 1.48)	1.58 (1.22, 2.05)	2.02 (1.52, 2.68)
Sodium	reference	1.07 (0.82, 1.38)	1.28 (1.01, 1.64)	1.92 (1.49, 2.48)	2.40 (1.83, 3.16)
BUN	reference	0.93 (0.71, 1.21)	0.94 (0.73, 1.21)	1.06 (0.79, 1.42)	1.04 (0.73, 1.48)
			Last		
Hemoglobin	reference	0.97 (0.78, 1.21)	1.20 (0.96, 1.49)	1.36 (1.06, 1.74)	1.92 (1.46, 2.52)
Sodium	reference	1.03 (0.83, 1.29)	1.32 (1.07, 1.64)	1.60 (1.25, 2.04)	2.23 (1.70, 2.91)
BUN	reference	0.88 (0.70, 1.10)	0.95 (0.76, 1.19)	0.88 (0.67, 1.14)	1.12 (0.82, 1.53)

Primary covariates included age, race, gender, health insurance, diabetes, hypertension, smoke, history of coronary heart disease, heart failure type (preserved vs. reduced ejection fraction), and chronic obstructive pulmonary disease.

Hemoglobin, sodium, and BUN were modeled as a continuous variable.

**Table 4:** Adjusted odds ratio of mortality outcomes according to BUN categories.

Models	BUN categories based on percentiles corresponding to clinical eGFR categories				
			Worst, range		
	<21	21–28	29–41	41–64	≥65
	n = 1032	n = 1053	n = 1328	n = 1187	n = 775
In-hospital	reference	0.72 (0.35–1.48)	1.74 (0.95–3.18)	3.08 (1.60–5.90)	7.18 (3.54–14.57)
28-day	reference	0.79 (0.49–1.28)	1.07 (0.68–1.69)	2.51 (1.53–4.12)	4.73 (2.68–8.34)
1-year	reference	1.18 (0.91–1.53)	1.32 (1.01–1.73)	2.12 (1.55–2.88)	4.39 (3.04–6.35)
			Last, range		
	<21	21–28	29–38	39–57	≥58
	n = 1652	n = 1186	n = 985	n = 940	n = 610
In-hospital	reference	0.70 (0.40–1.24)	1.34 (0.78–2.31)	3.32 (1.99–5.53)	10.35 (5.85–18.32)
28-day	reference	0.81 (0.53–1.23)	1.38 (0.91–2.10)	3.37 (2.21–5.15)	7.90 (4.89–12.78)
1-year	reference	1.09 (0.87–1.37)	1.48 (1.16–1.89)	2.25 (1.72–2.93)	5.20 (3.73–7.25)

Adjusted for primary covariates (age, race, gender, health insurance, diabetes, hypertension, smoke, history of coronary heart disease, heart failure type [preserved vs. reduced ejection fraction], and chronic obstructive pulmonary disease) and eGFR (as a continuous variable).

BUN was divided into five groups to approximately match with percentiles corresponding to clinical categories of eGFR in Table 2. The four cutoff points for worst and last BUN corresponded to 19^th^, 39^th^, 63^rd^, and 86^th^ percentiles and 31^st^, 53^rd^, 71^st^, and 89^th^ percentiles, respectively, whereas those for clinical categories of worst and last eGFR in Tables were 19^th^, 38^th^, 63^rd^, and 85^th^ percentiles and 31^st^, 52^nd^, 73^rd^, and 88^th^ percentiles, respectively.

Finally, we evaluated whether the use of evidence-based HF medications at discharge varies across last eGFR categories among those who were discharged alive within 28 days (unweighted n = 4,765 corresponding to weighted 21,941cases) (Table 5). Patients with reduced eGFR were less likely to receive renin-angiotensin system inhibitors. The use of beta-blockers and diuretics were largely comparable across eGFR categories except for diuretics in patients with kidney failure (eGFR <15 ml/min/1.73m^2^). However, the association of low eGFR with mortality risk was consistent even after additionally adjusting for or stratified by the use of HF medications (data not shown).

**Table 5:** Use of evidence-based HF medications across last eGFR categories among patients who were discharged alive within 28 days.

	eGFR categories				
Medication	≥60	45–59	30–44	15–29	<15/dialysis
ACE inhibitors	3484 (52.2)	2422 (48.3)	2090 (40.2)	826 (24.1)	712 (31.7)
ARB	844 (12.7)	685 (13.7)	667 (12.8)	360 (10.5)	210 (9.4)
BETA blocker	4764 (71.4)	3611 (72.0)	3896 (74.9)	2649 (77.4)	1761 (78.4)
Diuretics	5205 (78.0)	4261 (84.9)	4315 (82.9)	2641 (77.1)	1037 (46.2)

Values are N (% in each eGFR category)

## Discussion

In this study based on the ARIC Community Surveillance, we confirmed that reduced eGFR is prevalent (~70% with last eGFR <60 ml/min/1.73m^2^ and ~30% with last eGFR <30 ml/min/1.73m^2^) in ADHF patients and confers mortality risk, particularly below eGFR <30 ml/min/1.73m^2^. This association remained significant after adjustment for potential confounders (e.g., diabetes and hypertension) and mediators (hemoglobin and sodium). Its contribution to poor prognosis tended to be more evident for shorter term (in-hospital or 28-day mortality) than 1-year outcome. Although as previously reported we confirmed that some evidence-based HF medications were less likely to be used among those with reduced eGFR, the relationship between low eGFR and mortality was not necessarily altered even when we accounted for these medications. As previously reported [30], of note, BUN, another kidney function marker, demonstrated a stronger association with poor prognosis compared to eGFR in ADHF patients.

The prevalence of reduced eGFR including severe stages among ADHF patients was higher in our study than in previous studies using eGFR or creatinine clearance as a marker of kidney function (~75% vs. 26%-64% for eGFR or creatinine clearance <60 and ~30% vs. <20% for eGFR <30 ml/min/1.73m^2^) [7, 10, 20]. There seem to be a few potential reasons behind this observation. Some of the previous studies were clinical trials and excluded those with reduced eGFR (or elevated serum creatinine) [7, 10]. Also, those studies included HF patients controlled in outpatient settings. In this context, it is important that our estimates were even higher than that from a large registry study of HF hospitalizations, ADHERE (64% with eGFR <60 ml/min/1.73m^2^ and 8% with <30 ml/min/1.73m^2^) [20]. ADHERE used serum creatinine at admission of ADHF, and it is possible that their serum creatinine concentrations were diluted as a result of fluid congestion in this population. Also, this registry relied on ICD codes for identifying HF and thus might include some stable chronic HF cases. Moreover, ADHERE obtained data between 2001 and 2004 whereas our study are more contemporary (2005 through 2011), and the prevalence of CKD in the community is estimated to be rising [31].

As previously reported [30], we confirmed that BUN is a stronger predictor of mortality than creatinine-based eGFR in ADHF patients. Actually, a recent systematic review reveals that BUN is one of the strongest predictors of poor prognosis in this clinical population [32]. BUN is a well-known marker of kidney function, but recent investigations highlight that BUN also reflects activation of neurohumoral axis, an important pathophysiological pathway in ADHF [33]. Also, BUN is known to reflect decreased kidney perfusion and thus may indicate cardiac low output or the need of extensive diuresis. Nevertheless, in terms of risk prediction among ADHF patients, taken previous and our results together, it seems reasonable to prioritize BUN over creatinine-based eGFR. However, eGFR should still be the first choice for assessing kidney function itself, as recommended in clinical guidelines, for drug dose adjustment and kidney function monitoring over the clinical course [23].

Given that reduced eGFR is consistently associated with high mortality in various populations [1–6], it is not surprising that reduced eGFR confers mortality risk in ADHF patients as well. Potential mechanisms linking kidney dysfunction to mortality risk include volume overload, oxidative stress, inflammation, and uremic toxins [34]. Also, poor control of traditional risk factors such as hypertension in patients with kidney disease is considered as an important contributor [34]. It has been demonstrated that patients with CKD are less likely to receive evidence-based therapy for cardiovascular disease management [29], and we confirmed this pattern for renin-angiotensin system inhibitors in our study. However, the relationship between kidney dysfunction and mortality was consistent even after accounting for the use of HF medications. Nevertheless, close attention to the quality of evidence-based HF management is warranted among HF patients with kidney dysfunction [29]. Also, as aforementioned, clinical trials frequently exclude patients with reduced kidney function [7, 10] and thus investigations of established HF medications specifically in HF patients with reduced kidney function would be needed.

Our study has several limitations. First, serum creatinine data at admission were not specifically abstracted, and thus we could not dissect impact of eGFR at admission and change in eGFR from admission [18, 35]. However, consistent findings regardless of when worst and last eGFRs were used support robustness of our findings. Second, we did not have information on whether the measurement of serum creatinine was standardized in relevant hospitals in the four ARIC communities. Third, we focused on kidney function in this study as we did not have data on albuminuria, a marker of kidney damage and the other key marker do define and stage CKD [23]. Thus, studies taking into account albuminuria in ADHF patients are warranted, particularly since close relationship between albuminuria and incident HF has been reported [36, 37]. Finally, we cannot deny the possibility of residual confounding, as true in any observation studies.

In conclusion, severely reduced kidney function (eGFR <30 ml/min/1.73m^2^) is found in ~30% of ADHF patients in the community and confers mortality risk (1 year mortality risk greater than 40% vs. ~25% if eGFR ≥60 ml/min/1.73m^2^) beyond potential confounders. In terms of mortality prediction, another kidney function parameter, BUN, seems superior to creatinine-based eGFR, probably due to its property reflecting both kidney and non-kidney pathophysiological pathways. These observations suggest that close attention to ADHF patients with kidney dysfunction is warranted.

## Supporting information

S1 Table Baseline characteristics according to eGFR categories based on the worst creatinine levels during ADHF hospitalization. (DOCX)
