# Supplementary material for: Community burden and prognostic impact of reduced kidney function among patients hospitalized with acute decompensated heart failure: The Atherosclerosis Risk in Communities (ARIC) Study Community Surveillance
Source: PLoS One. 2017 Aug 9;12(8):e0181373. doi: 10.1371/journal.pone.0181373 (PMC5549913; doi:10.1371/journal.pone.0181373)
Supplement: S1 Table — (DOCX) [file pone.0181373.s001.docx]

S1 Table. Baseline characteristics according to eGFR categories based on the worst creatinine levels during ADHF hospitalization

| **Baseline characteristics** | **eGFR:60+** | **eGFR:60-45** | **eGFR:45-30** | **eGFR:30-15** | **eGFR<15** |
| --- | --- | --- | --- | --- | --- |
| Weighted N | 4365 (17.5) | 4661 (18.7) | 6599 (26.5) | 5798 (23.3) | 3510 (14.1) |
| Age | 71 (22.4) | 75 (22.8) | 77 (22.5) | 79 (21.3) | 74 (20.9) |
| Female | 1984 (45.5) | 2423 (52.0) | 3589 (54.4) | 3364 (58.0) | 1804 (51.4) |
| African-Americans | 1644 (37.7) | 1239 (26.6) | 1573 (23.8) | 1527 (26.3) | 1285 (36.6) |
| Smoker | 924 (21.2) | 722 (15.5) | 759 (11.5) | 569 (9.8) | 383 (10.9) |
| Hypertension | 3506 (80.3) | 3746 (80.4) | 5751 (87.2) | 4957 (85.5) | 3104 (88.4) |
| Diabetes | 1894 (43.4) | 1892 (40.6) | 2934 (44.5) | 2970 (51.2) | 2236 (63.7) |
| Health Insurance |  |  |  |  |  |
| Medicare/Medicaid | 1202 (29.7) | 1292 (29.0) | 1842 (28.8) | 1659 (29.6) | 1234 (36.4) |
| Private | 2848 (70.3) | 3162 (71.0) | 4558 (71.2) | 3947 (70.4) | 2153 (63.6) |
| History of HF | 2789 (63.9) | 3180 (68.2) | 4728 (71.6) | 4276 (73.8) | 2701 (76.9) |
| Prior HF hospitalization | 1401 (32.1) | 1545 (33.2) | 2429 (36.8) | 2062 (35.6) | 1432 (40.8) |
| Prior HF treat | 2227 (51.0) | 2762 (59.3) | 3942 (59.7) | 3726 (64.3) | 2199 (62.7) |
| Ejection fraction |  |  |  |  |  |
| Preserved | 1658 (42.3) | 1770 (41.9) | 2674 (44.0) | 2369 (45.4) | 1400 (46.2) |
| Reduced | 2260 (57.7) | 2456 (58.1) | 3402 (56.0) | 2850 (54.6) | 1632 (53.8) |
| COPD | 1715 (39.3) | 1691 (36.3) | 2420 (36.7) | 1893 (32.7) | 1133 (32.3) |
| Coronary Disease | 1896 (43.4) | 2117 (45.4) | 3143 (47.6) | 2664 (46.0) | 1684 (48.0) |
| Hemoglobin | 12 (1.8) | 12 (2.0) | 11 (1.8) | 11 (1.6) | 11 (1.7) |
| Sodium | 138 (3.7) | 139 (3.7) | 139 (3.9) | 139 (4.4) | 138 (4.5) |
| BUN | 18 (8.3) | 23 (9.2) | 30 (13.7) | 45 (20.6) | 52 (28.3) |
| Systolic BP | 145 (30.4) | 145 (32.5) | 141 (33.3) | 139 (33.3) | 142 (38.2) |
| Diastolic BP | 82 (19.7) | 80 (19.2) | 77 (20.2) | 74 (19.6) | 75 (20.9) |

Values are mean (SD) and n (%).
